# Baseline characteristics and survival of patients of idiopathic pulmonary fibrosis: a longitudinal analysis of the Swedish IPF Registry

**DOI:** 10.1186/s12931-021-01634-x

**Published:** 2021-02-05

**Authors:** Jing Gao, Dimitrios Kalafatis, Lisa Carlson, Ida H. A. Pesonen, Chuan-Xing Li, Åsa Wheelock, Jesper M. Magnusson, C. Magnus Sköld

**Affiliations:** 1 Respiratory Medicine Unit, Department of Medicine, Karolinska Institute, Solna, Solnavägen 30, 17176 Stockholm, Sweden; 2 Department of Respiratory Medicine and Allergy, Karolinska University Hospital, Stockholm, Sweden; 3 Department of Internal Medicine/Respiratory Medicine and Allergology, Institute of Medicine, Sahlgrenska University Hospital, Gothenburg, Sweden

**Keywords:** Idiopathic pulmonary fibrosis, Disease severity, Phenotype, Anti-fibrotic treatment, Survival

## Abstract

**Background:**

Observational data under real-life conditions in idiopathic pulmonary fibrosis (IPF) is scarce. We explored anti-fibrotic treatment, disease severity and phenotypes in patients with IPF from the Swedish IPF Registry (SIPFR).

**Methods:**

Patients enrolled between September 2014 and April 2020 and followed ≥ 6 months were investigated. Demographics, comorbidities, lung function, composite variables, six-minute walking test (6MWT), quality of life, and anti-fibrotic therapy were evaluated. Agreements between classification of mild physiological impairment (defined as gender-age-physiology (GAP) stage 1) with physiological and composite measures of severity was assessed using kappa values and their impact on mortality with hazard ratios. The factor analysis and the two-step cluster analysis were used to identify phenotypes. Univariate and multivariable survival analyses were performed between variables or groups.

**Results:**

Among 662 patients with baseline data (median age 72.7 years, 74.0% males), 480 had a follow up ≥ 6 months with a 5 year survival rate of 48%. Lung function, 6MWT, age, and BMI were predictors of survival. Patients who received anti-fibrotic treatment ≥ 6 months had better survival compared to untreated patients [*p* = 0.007, HR (95% CI): 1.797 (1.173–2.753)] after adjustment of age, gender, BMI, smoking status, forced vital capacity (FVC) and diffusion capacity of carbon monoxide (DLCO). Patients with mild physiological impairment (GAP stage 1, composite physiological index (CPI) ≤ 45, DLCO ≥ 55%, FVC ≥ 75%, and total lung capacity (TLC) ≥ 65%, respectively) had better survival, after adjustment for age, gender, BMI and smoking status and treatment. Patients in cluster 1 had the worst survival and consisted mainly of male patients with moderate-severe disease and an increased prevalence of heart diseases at baseline; Cluster 2 was characterized by mild disease with more than 50% females and few comorbidities, and had the best survival; Cluster 3 were younger, with moderate-severe disease and had few comorbidities.

**Conclusion:**

Disease severity, phenotypes, and anti-fibrotic treatment are closely associated with the outcome in IPF, with treated patients surviving longer. Phenotypes may contribute to predicting outcomes of patients with IPF and suggest the patients’ need for special management, whereas single or composite variables have some limitations as disease predictors.

## Introduction

Idiopathic pulmonary fibrosis (IPF) is a chronic, progressive fibrosing interstitial lung disease (ILD) of unknown cause[1–3].The disease is characterized by an aberrant accumulation of fibrotic tissue in the lung parenchyma, resulting in extensive alterations of lung structure and function and leads finally to respiratory failure and death [2, 4, 5]. Long-term observational studies in clinically diverse IPF populations from all over the world are increasing [1, 6–15] and provide us with important information on disease behaviour, management, and effectiveness of approved treatments.

Forced vital capacity (FVC), diffusion capacity for carbon monoxide (DLCO), composite physiological index (CPI) [7, 16] and GAP (gender, age and physiology) stage have been used to define the severity of IPF and to predict mortality[7, 17, 18]. In a recent study, a six-minute walking test (6MWT) was proved to be important predictors for survival [19]. GAP stage 1 has been commonly used as a mild physiological impairment criterion [7]. However, the impact of these physiological variables on disease progression and mortality in patients with mild or more advanced disease is largely unknown. Furthermore, we have previously indicated potential gender differences in patients with IPF [20]. Thus, an unsupervised cluster analysis may provide novel insights into the phenotypes of IPF with potential prognostic significance. Progress in the management of IPF has been made with the introduction of two antifibrotics, pirfenidone and nintedanib, which have been shown to reduce the rate of disease progression [21, 22]. However, strict inclusion and exclusion criteria in clinical trials may limit the generalizability of the results in real clinical settings. For instance, patients with comorbidities, lower lung function, and concomitant medications have been commonly excluded from participation in randomized clinical trials [23–25]. Therefore, many questions remain about the generalizability of these findings to a wider IPF population.

Given the lack of knowledge on disease course and mid- to long-term outcomes in IPF, our aims were to explore characteristics, disease severity, phenotype, and anti-fibrotic treatments in patients with IPF under real-life conditions and to assess associations to mortality. We also wanted to ascertain whether further characterization may help patients with IPF and aid the development of personalized management and/or therapy. Additionally, we compared our data with other registries to highlight clinical and geographical variability.

## Patients and methods

### Study population

The Swedish IPF Registry (SIPFR) is a nationwide registry collecting comprehensive longitudinal data of IPF patients and implemented in 22 respiratory medicine units across Sweden [13, 19, 20]. The SIPFR also includes patients diagnosed before the registry was launched in 2014. The registry relies on a web-based platform (Granitics Unify Med, Granitic Ltd, Espoo, Finland) which allows secure data collection at each respective center. Data entries are made by nurses and physicians at each site, and the quality of the data is evaluated and improved by source data verification performed by the registry coordinator (LC). To be eligible for inclusion in the registry, the patient has to have a confirmed diagnosis of IPF according to the national and international guidelines [13, 26, 27] by a specialist in respiratory medicine either at a university hospital or a local hospital. The registry applies no explicit exclusion criteria, thereby reducing selection bias. We included all patients enrolled in the registry from Sep 2014 until April 2020, and the patients followed ≥ 6 months were enrolled in survival analyses. The outcome of death was defined as patients dying or receiving a lung transplant during the observation period. Patients who were alive at the last visit date during the follow-up period of this study were censored and classified as survivors. The primary survival time was calculated from the enrolment date, with baseline data. Secondary survival time was calculated from the diagnosis date, without matched baseline data.

### Variables

Data covering demographics, self-reported comorbidities, lung function, 6MWT, radiology, quality of life (assessed with the King's Brief Interstitial Lung Disease Questionnaire (K-BILD)) and anti-fibrotic therapy were included [13]. Charlson Comorbidity Index (CCI) was calculated with an ad hoc modified formula, i.e. coronary artery disease, other cardiovascular diseases, diabetes, arterial hypertension, chronic obstructive pulmonary disease, and acid reflux gave one point each and history of cancer gave two points. The composite physiologic index (CPI) was calculated using the formula: CPI = 91.0—(0.65 × % predicted DLCO)—(0.53 × % predicted FVC) + (0.34 × % predicted FEV_1_). The gender-age-physiology (GAP) index was extrapolated for each patient with available data in the registry using the variables of the scoring system combining gender, age, and lung physiology (FVC and DLCO) and classified as GAP stage I (0–3 points), GAP stage II (4–5 points), or GAP stage III (6–8 points). Patients with smoking history included ex-smokers and/or current smokers. Patients were considered as "incident” cases if diagnosed within 6 months from inclusion, while patients with a diagnosis of more than 6 months from inclusion were considered as "prevalent". Each patient's IPF diagnosis was evaluated by "clinic radiological", "thoracoscopic biopsy", "open lung biopsy", or " multidisciplinary conference". Exposure was ascertained by the answers to self-reported questions, such as microbes, particles from the atmosphere, irritants, pollutants, allergens, and pathogens [28]. Data collected at 6 months prior to or after the consent date in this study was considered as baseline data.

### Anti-fibrotic therapy

Treatment status was classified into an anti-fibrotic treatment group (receiving anti-fibrotic therapy after diagnosis ≥ 6 months) and untreated group (no treatment or anti-fibrotic therapy after diagnosis < 6 months) [10]. The anti-fibrotic treatment group was further classified into three groups: (1) patients only treated with nintedanib ≥ 6 months; (2) patients only treated with pirfenidone ≥ 6 months; (3) patients switched between pirfenidone (≥ 6 months) and nintedanib (≥ 6 months).

### Classification of disease severity using different mild definitions

Disease severity was evaluated as mild and moderate to severe physiological impairment using different criteria [7]. We compared GAP criteria for mild physiological impairment (GAP stage 1) against other proposed criteria: FVC ≥ 75% (exploratory analysis for ≥ 90%,  ≥ 80%, and ≥ 70%); DLCO ≥ 55% (exploratory analysis of  ≥ 60%,  ≥ 50%, and ≥ 45%), TLC ≥ 65% (exploratory analysis for  ≥ 75%,  ≥ 70%, and  ≥ 60%), and CPI ≤ 45 (exploratory analysis CPI ≤ 30, CPI ≤ 40, CPI ≤ 50) exploring the agreement in classifications and relationship with disease outcomes (data not shown).

### Cluster analysis on baseline SIPFR data with disease severity

A two-step cluster analysis was used to differentiate the patients into distinct phenotypes. Input variables for the cluster analysis were based on the basis of factor analysis, including baseline characteristics (age, gender, BMI), comorbidities (the number of comorbidities, CCI, acid reflux and cardiovascular diseases), and severity (cut-off level: GAP stage 1, FVC ≥ 75%, DLCO ≥ 55%, TLC ≥ 65% and CPI ≤ 45). The Kaiser–Meyer–Olkin (KMO) value of the scale (> 0.6) and the Bartlett test value of sphericity (*p* < 0.05) were used to determine the sampling adequacy for factor analysis. Cluster analysis was carried out by using a two steps process [29]. First, the number of clusters were pre-evaluated by Ward hierarchical cluster analysis and factor analysis. Then, the K-means cluster analysis was carried out by using the pre-specified number of clusters. The stepwise discriminant analysis was performed to identify variables discriminating amongst the clusters. For validation, we carried out the leave-one-out method to ensure the stability and repeatability of the cluster model.

### Other statistical analyses

Descriptive analysis was performed with medians with interquartile ranges (IQR), or mean ± standard deviation (SD) for continuous variables, and counts with percentages for categorical variables. Missing data, primarily due to data not being registered, was not estimated but was removed from the denominator in calculation. Comparisons between groups were performed using t-test, ANOVA, Mann–Whitney U test, Chi-squared test, or pairwise comparison as appropriate. Univariable and multivariable Cox regression models were performed to investigate the relationships between baseline variables. All models were examined for assumptions of normality of the residuals and homogeneity of variance by examination of residual plots. Kaplan–Meier estimates and a log rank test for mortality were performed to calculate mortality by selected variables. The log-rank test was used to test the differences in survival between the two groups of patients. Comparisons with other IPF cohorts are descriptive and based on published data [1, 6, 9, 10]. All statistical analyses were performed using IBM’s SPSS Statistics version 21 (SPSS, Chicago, IL, USA), Stata 13.1 software package (StataCorp LP, College Station, TX, USA), and GraphPad Prism version 6.0 (GraphPad Software, San Diego, CA, USA). We considered *p* < 0.05 as statistically significant.

## Results

### SIPFR cohort

**Fig. 1:**
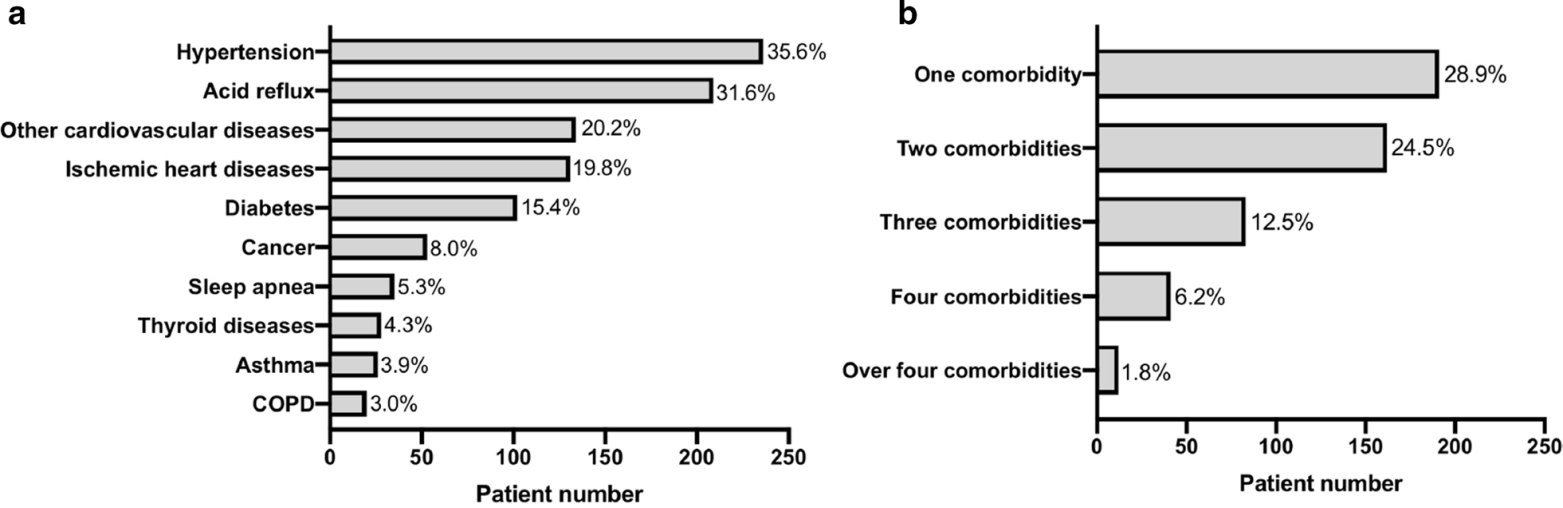
Prevalence of comorbidities in the SIPFR. The number and percent of **a** single comorbidity, **b** the combination of comorbidities. *COPD* chronic obstructive pulmonary disease

Included patients (n = 662, median age 72.7 years, males 74.0%) were collected between Sep. 2014 and Apr. 2020 (Table 1). Almost two thirds of patients reported a history of smoking, with approximately 60% of patients being ex-smokers, and 24 patients (4%) current smokers (Table 1). The time from IPF diagnosis to enrolment was 2 months. GAP stage was available for 384 patients, and the distribution of GAP stage was I (51.0%), II (40.9%) and III (8.1%). The median value of CCI was 4. The most frequently reported group of comorbidities were cardiovascular diseases, with 54.5% of patients reporting at least one cardiovascular disease (these included hypertension 35.6% of all patients, other cardiovascular diseases 31.6%, and ischaemic heart disease 20.2%) (Fig. 1). Approximately over 70% patients reported at least one comorbidity, and more than 40% of patients had two or three comorbidities at baseline (Fig. 1). According to the primary survival timeline, 480 patients were followed ≥ 6 months from enrolment date (median (interquartile range) 28 (15–46.5) months), while the secondary survival timeline, included 540 patients who had been followed ≥ 6 months from diagnosis date (20 (12–32) months).

**Table 1:** Baseline characteristics of the SIPFR

Variable	Total, n	Value
Age, years, at enrolment into the registry	662	72.7 (68.0–78.0)
Age, years, at diagnosis	651	72.0(67.0–77.0)
The time from IPF diagnosis to enrolment, months	651	2 (0–15)
Gender (male, n, %)	662	490 (74.0)
Basis of diagnosis	662	
Multidiscipline conference (MDC) diagnosis		275 (41.5)
Clinic radiological		587 (88.7)
Thoracoscopic biopsy		38 (5.7)
Open lung biopsy		31 (4.1)
UIP status (confirmed and possible UIP, n, %)	662	434 (65.5)
Incident IPF, (n, %)	662	422 (64.8)
Exposure	662	208 (31.4)
Smoking history (yes, n, %)	662	429 (64.8)
Ex-smokers		405 (61.2)
Current-smokers		24 (3.6%)
BMI	595	26.6 (24.2–29.4)
Underweight (< 18.5 kg·m^2^, %)		5 (0.8)
Normal weight (≥ 18.5– ≤ 25 kg·m^2^, %)		196 (29.6)
Overweight (> 25– ≤ 30 kg·m^2^, %)		266 (40.2)
Obesity (> 30 kg·m^2^, %)		128 (19.3)
Physiology*		
FEV1, % predicted	540	78.0 (66.0–90.0)
FVC, % predicted	507	71.0 (61.0–85.0)
FEV1/FVC, %	512	0.81 (0.76–0.86)
DLCO, % predicted	394	47.0 (37.0–56.0)
TLC, % predicted	361	66.0 (57.0–74.0)
CPI	361	47.2 (40.5–55.3)
GAP stage 1, (n, %)	384	157 (40.9)
Stage 2, (n, %)	384	196 (51.0)
Stage 3, (n, %)	384	31 (8.1)
Six-minute walk test*		
6MWD (m)	375	430 (363–500)
L-SpO2 (%)	378	87.0 (82.0–91.0)
Quality of life*		
K-BILD	385	55.0 (48.0–62.0)
Comorbidities (yes, n%)	662	488 (73.7)
CCI		4 (3–5)
Anti-fibrotic therapy (yes, n %)	662	360 (54.4)
Death or transplant status (yes, n %)	662	218 (32.9)

Data are presented as median (25th percentile-75th percentile) unless otherwise indicated. *SIPFR* the Swedish idiopathic pulmonary fibrosis registry, *BMI* body mass index; *MDC* multidisciplinary conference (radiological, histopathological and clinical) panel evaluation, *UIP pattern* confirmed and possible usual interstitial pneumonia; *FEV*_*1*_ forced expiratory volume in 1 s, *FVC* forced vital capacity, *DLCO* diffusing capacity of carbon monoxide, *TLC *Total lung capacity, *CPI* composite physiological index, *GAP* gender-age-physiology index for IPF, *K-BILD* King's brief interstitial lung disease health status questionnaire, *6MWT* 6-min walking test, *6MWD* 6-min walking distance during the 6MWT, *L-SpO*_*2*_ lowest oxygen saturation during 6MWT, *CCI* Charlson Comorbidity Index, *Performed or data collected 6 months before or after registry inclusion

**Fig. 2:**
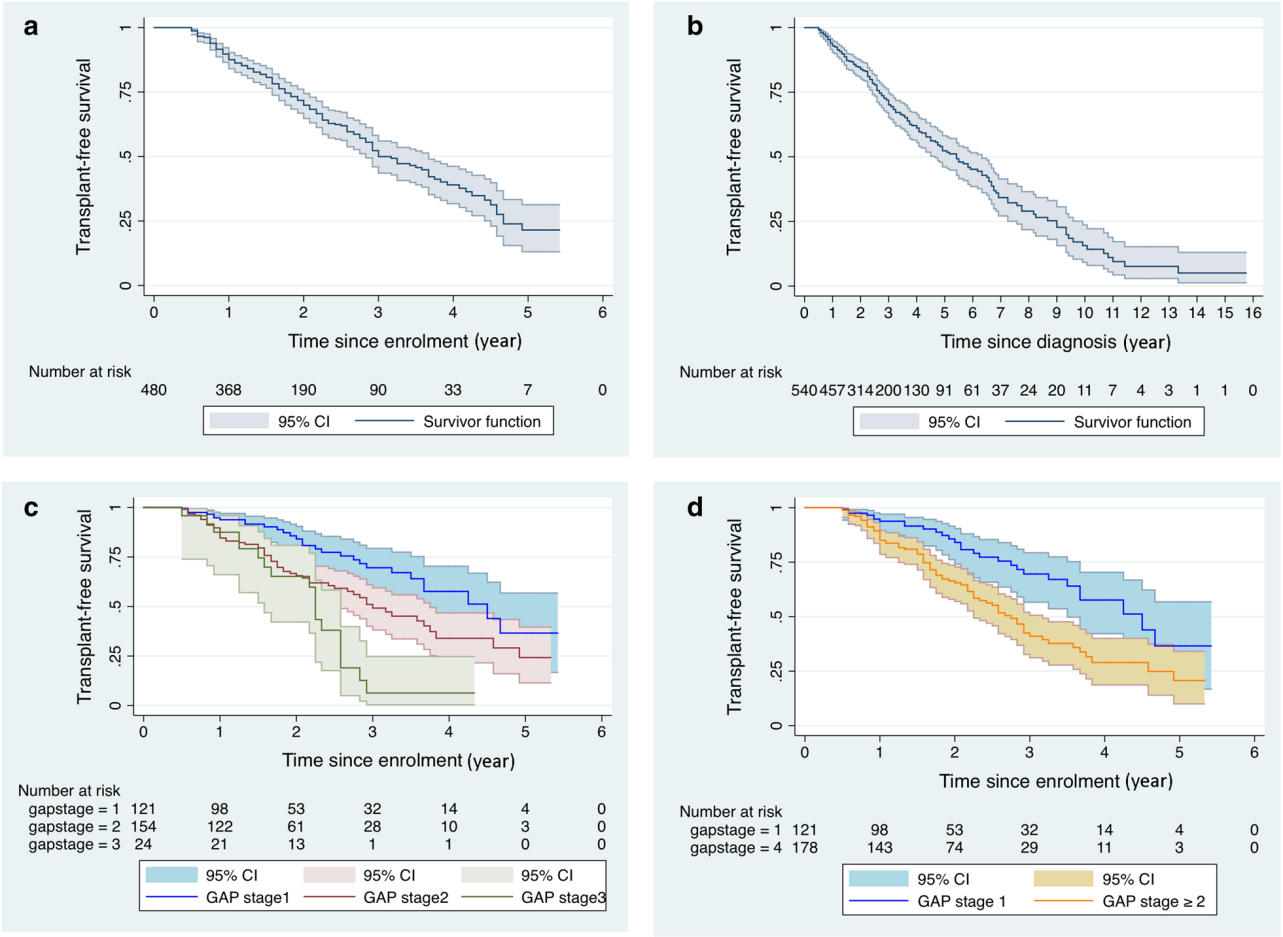
Kaplan–Meier analysis for survival in the cohort and in GAP stages. Kaplan–Meier analysis for mortality in the SIPFR cohort according to **a** time from the enrolment; **b** time from the diagnosis; **c** and **d** *GAP stage* GAP gender, age, physiology

During the follow-up time, 195 had died and 23 had undergone lung transplants. The increasing cumulative rate of death from the diagnosis date in one to five years was 7, 16, 30, 39, and 48%, respectively. The cumulative rate from the enrolment date in one to five years was 12, 32, 50, 62 and 78% (Fig. 2a). Figure 2 displays the different cumulative rates of death according to the GAP stage. A trend of shorter survival in male patients compared to women was observed (median: 35.0 vs. 44 months, log rank p = 0.067). In the univariate Cox analysis of baseline factors, decreased lung function, six-minute walking distance (6MWD), K-BILD, BMI, age, smoking history, CPI and GAP, were significant predictors of mortality (Table 2).

**Table 2:** Univariable Cox analysis for survival

Variable	n	HR	95% CI	*p*-value
Age, years, at enrolment into the registry	480	1.027	1.006–1.049	0.012
Male versus female	480	1.373	0.973–1.936	0.071
BMI, per kg·m^2^	450	0.948	0.911–0.986	0.008
Ex/current smoker versus never-smoker	480	1.43	1.052–1.944	0.023
CCI index	480	1.039	0.944–1.144	0.435
Comorbidities number	480	0.989	0.881–1.109	0.848
Heart diseases	480	1.134	0.842–1.529	0.407
Acid reflux	480	0.760	0.550–1.050	0.096
FVC % predicted	385	0.972	0.962–0.983	< 0.001
DLCO % predicted	310	0.957	0.943–0.971	< 0.001
TLC % predicted	282	0.952	0.936–0.969	< 0.001
CPI	282	1.064	1.043–1.085	< 0.001
GAP stage	290	1.943	1.448–2.607	< 0.001
6MWD, m	284	0.996	0.995–0.998	< 0.001
LpSO2, %	284	0.934	0.904–0.965	< 0.001
K-BILD	317	0.965	0.947–0.983	< 0.001

*IPF* Idiopathic pulmonary fibrosis, *HR* hazard ratios, *BMI* Body mass index, *CCI* Charlson Comorbidity Index, *FVC* Forced vital capacity, *DLCO* Diffusing capacity of carbon monoxide, *TLC* total lung capacity, *CPI* composite physiological index, *GAP* gender-age-physiology index for IPF, *K-BIL* King's brief interstitial lung disease health status questionnaire, *6MWD* 6-min walking distance during 6MWT, *L-SpO*_*2*_ Lowest oxygen saturation during 6MWT

### Comparison with other IPF-registries

Comparison of the SIPFR with the Australian IPF Registry [6] Finnish IPF Registry [10] (FinnishIPF, n = 453), the German INSIGHTS-IPF Registry [9] (INSIGHTS, n = 623) and European IPF registry [1] (EurIPFreg, n = 525) is outlined in Table 3. Age and gender distributions were similar in all registries, whereas patients in SIPFR had lower BMI. Baseline lung functions in SIPFR were more preserved than in INSIGHTS- and EurIPF -registries, but worse than in AIPFR and the FinnishIPF. The 6MWD was similar in SIPFR and AIPFR, whereas the distance was greater than the one reported in the INSIGHTS- and EurIPF-registry. Only two registries presented data on TLC% at baseline. Swedish IPF registry presented a lower TLC% compared to EurIPFreg. The cumulative rate of death data from reports were available for SIPFR, AIPFR and FinnishIPF, with one-year mortality of 7% 5%, and 5%, respectively.

**Table 3:** Comparison of baseline characteristics on SIPFR to other published IPF registries

	Swedish	Australian	Finnish	INSIGHTS^9^	EurIPFreg^1^
	IPF	IPF^6^	IPF^10^		
Patients, n	662	647	453	623	525
Male (%)	74.0	67.7	65.1	77.2	73.7
Age	72.7 (7.5)	70.9 (8.5)	73.5 (9.0)	69.6 (8.7)	68.1 (11.1)
BMI kg·m^2^	27.0 (4.1)	28.7(4.8)	28.4 (5.2)	27.5 (4.1)	27.2 (4.6)
Ex-smokers (%)	61.2	71.7	48.0	60.4	65.4
FVC, % predicted	72.7 (17.1)	81.0 (21.7)	80.2 (18.0)	67.5 (17.8)	68.4 (22.6)
DLCO, % predicted	48.4 (14.7)	48.40%	55.6 (16.5)	35.6 (17.0)	42.1 (17.8)
GAP (stage 1, %)	40.9	46.2	54.1	20.2	n.a
TLC %	65.6 (12.7)	n.a	n.a	n.a	70.0 (38.4)
6MWD	420 (118)	420 (129)	n.a	272 (196)	388 (122)
The cumulative rate of death					
1 year (%)	7	5	5	n.a	n.a
2 year (%)	16	24	17	n.a	n.a
3 year (%)	30	37	30	n.a	n.a
4 year (%)	39	44	42	n.a	n.a
5 year (%)	48	n.a	55	n.a	n.a

Data are presented as mean with SD unless otherwise indicated. *IPF* idiopathic pulmonary fibrosis, *BMI* body mass index, *FVC* forced vital capacity, *DLCO* diffusing capacity of carbon monoxide, % of predicted; *TLC* total lung capacity, % of predicted, *GAP* gender-age-physiology index for IPF, *6MWD* 6 min walking distance during 6MWT, *n.a.* not available or not available at baseline report

### Anti-fibrotic therapy

**Fig. 3:**
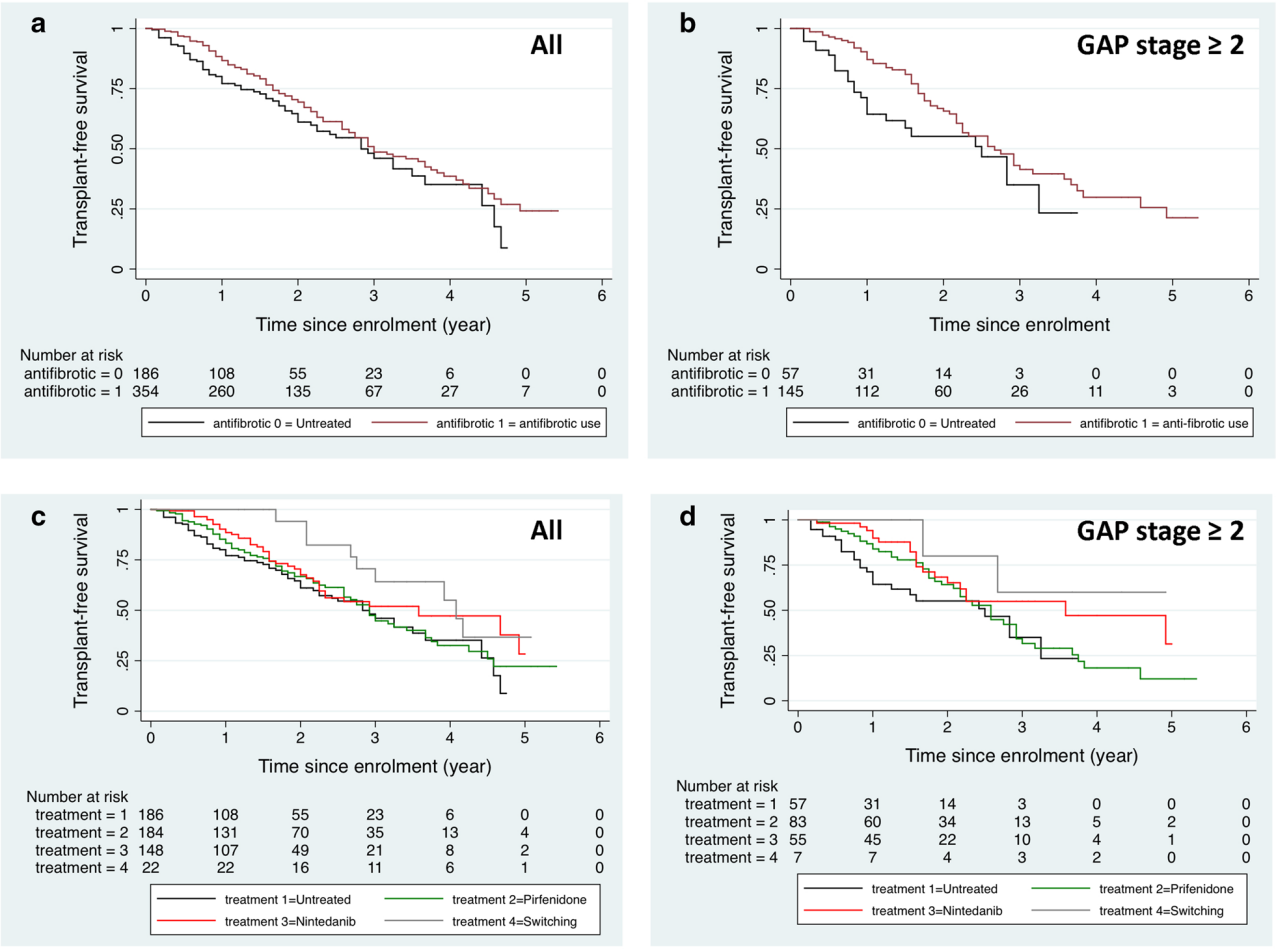
Kaplan–Meier analysis for survival in treatment. Kaplan–Meier analysis for mortality in the SIPFR cohort according to **a**, **b** patients with and without anti-fibrotic treatment in patients in all and GAP stage over 1; **c**, **d** patients with anti-fibrotic treatment (nintedanib, pirfenidone, switched treatment) and untreated in patients in all and GAP stage over 1

Among the 540 patients with a follow up of ≥ 6 months from diagnosis, 347 (64.3%) received anti-fibrotic treatment for ≥ 6 months from diagnosis date, either with pirfenidone or nintedanib (33.9% and 26.3% respectively). A minor group of patients (4.1%) had switched treatment. Patients on anti-fibrotic therapy were younger compared to those who did not receive treatment (*p* = 0.018, Table 4). The median age at diagnosis of the “switched” group, “pirfenidone treated” group, and “nintedanib treated” group were 67.0 years, 72.0 years, and 72.0 years, respectively. However, the difference in age at diagnosis was not statistically significant (*p* = 0.056). Two thirds of patients (n = 218) had a smoking history (Table 4), with 3 current smokers receiving pirfenidone, 2 current smokers nintedanib, and 1 current smoker had switched treatment. The median age at diagnosis of the “switched” group, “pirfenidone treated” group, and “nintedanib treated” group were 67.0 years, 72.0 years, and 72.0 years, respectively. However, the difference in age at diagnosis was not statistically significant (p = 0.056). Two thirds of patients (n = 218) had a smoking history (Table 4), with 3 current smokers receiving pirfenidone, 2 current smokers nintedanib, and 1 current smoker had switched treatmentFVC % predicted and GAP stage did not differ between patients treated with anti-fibrotic and those who did not receive treatment (Table 4). GAP stage did not differ between nintedanib and pirfenidone treated patients (*p* = 0.807 and *p* = 0.116, respectively). Kaplan–Meier analysis showed improved survival in patients on anti-fibrotic therapy compared to untreated patients in all and in patients with GAP stage ≥ 2 ((log rank *p* = 0.037 and *p* = 0.034, Fig. 3a, b). When we separately analyzed the two anti-fibrotic drugs, we found that patients receiving nintedanib had better survival compared to untreated patients in all and in patients with GAP stage ≥ 2 (log rank *p* = 0.034 and p = 0.025, respectively, Fig. 3c, d). In addition, patients switching treatment also had a better survival compared to untreated patients (log rank *p* = 0.026, Fig. 3c). In the multivariate Cox regression analysis, patients with anti-fibrotic treatment still had a better prognosis than those without (*p* = 0.007, HR (95% CI): 1.797 (1.173–2.753)) after adjustment of age, gender, BMI, smoking status, FVC%, and DLCO%.

**Table 4:** Baseline characteristics in treatment

	Untreated	Anti-fibrotic treatment				*P*-value*
		All	Pirfenidone	Nintedanib	Switched	
N	150	330	166	142	22	
Age, years, at enrolment into the registry	74.5 (70.0–79.0)	73.0 (67.0–77.0)	73.0 (68.0–78.0)	73.0 (67.0–78.0)	69.5 (64.0–73.0)	0.018
Age, years, at diagnosis	73.0 (69.0–78.0)	72.0 (66.0–77.0)	72.0 (67.0–77.0)	72.0 (66.0–77.0)	67.0 (63.0–70.0)	0.027
Gender (male, n, %)	100 (66.7)	242 (73.3)	128 (77.1)	98 (69.0)	16 (72.7)	0.135
Smoking history (yes, n, %)	99 (66.0)	218 (66.1)	118 (71.1)	88 (62.0)	12 (54.5)	0.99
BMI	26.2 (23.6–29.1)	27.1 (24.5–30.0)	26.9 (24.5–29.9)	27.2 (24.5–30.0)	27.9 (24.7–30.1)	0.052
FVC, % predicted	72.0 (62.0–86.0)	69.0 (60.0–82.0)	69.0 (60.0–80.0)	72.0 (60.5–85.0)	65.0 (61.0–77.0)	0.169
DLCO, % predicted	50.0 (42.0–59.5)	47.0 (37.0–56.0)	46.0 (36.0–56.0)	47.0 (37.0–56.0)	47.0 (41.0–56.0)	0.044
TLC, % predicted	66.0 (58.5–73.0)	65.0 (55.0–72.0)	64.0 (55.0–71.0)	65.0 (56.0–73.0)	66.0 (56.0–71.0)	0.072
CPI	44.1 (38.1–51.3)	48.9 (41.4–55.7)	49.7 (41.4–56.5)	48.9 (41.7–55.3)	46.2 (37.4–55.4)	0.002
GAP stage	2 (1–2)	2 (1–2)	2 (1–2)	2 (1–2)	1 (1–2)	0.426
6MWD (m)	418 (360–477)	430 (365–503)	420 (351–495)	435 (385–498)	473 (393–536)	0.211

Data are presented as median (25th percentile-75th percentile) unless otherwise indicated. *IPF* idiopathic pulmonary fibrosis; *BMI* body mass index, *FVC* forced vital capacity; *DLCO* diffusing capacity of carbon monoxide, *TLC* total lung capacity, *CPI* composite physiological index, *GAP* gender-age-physiology index for IPF, *6MWD* 6 min walking distance during 6MWT; *P-value was compared between untreated group and anti-fibrotic treatment group

### Classification of disease severity

Altogether 243 patients were followed ≥ 6 months, after exclusion of patients with missing data on FVC%, DLCO%, TLC%, CPI, and GAP stage. Mild physiological impairment defined by GAP stage 1 had a good agreement with CPI ≤ 45 (kappa value (k) = 0.62), and moderate agreement with DLCO ≥ 55% (k = 0.58), FVC ≥ 75% (k = 0.50), and TLC ≥ 65% (k = 0.47). Mild physiological impairment at baseline (DLCO ≥ 55%, TLC ≥ 65%, CPI ≤ 45, FVC ≥ 75% and GAP stage 1, respectively) was predictive of better survival compared to patients with moderate-severe disease in univariable analysis, as well as multivariable Cox analysis after adjustment of age, gender, BMI, smoking status and anti-fibrotic use (Table 5).

**Table 5:** Crude and adjusted hazard ratios (HR) of mortality for the disease severity in Cox regression model

Variables	Number (%)	Crude HR (95% CI)	Adjusted HR (95% CI)^#^
GAP stage			
≥ 1	143 (59)	2.415 (1.511–3.860)***	2.258 (1.339–3.808)**
1	110 (41)	Reference	Reference
FVC% predicted			
< 75%	133 (55)	1.741 (1.131–2.680)*	2.118 (1.341–3.346)**
≥ 75%	110 (45)	Reference	Reference
DLCO% predicted			
< 55%	159 (65)	2.499 (1.455–4.290)**	2.646 (1.506–4.650)**
≥ 55%	84 (35)	Reference	Reference
TLC % predicted			
< 65%	105 (43)	2.327 (1.523–3.554)***	2.195 (1.422–3.389)***
≥ 65%	138 (57)	Reference	Reference
CPI			
> 45	136 (56)	3.246 (1.986–5.303)***	3.619 (2.158–6-071)
≤ 45	107 (44)	Reference	Reference

*HR* hazard ratios, *GAP* gender-age-physiology index for IPF, *FVC* forced vital capacity, *DLCO* diffusing capacity of carbon monoxide, *TLC* total lung capacity, *CPI* composite physiological index. ^#^Multivariable model includes age, gender, BMI, smoking status and treatment *p < 0.05; **p < 0.01; and ***p < 0.001

### Cluster analysis

**Fig. 4:**
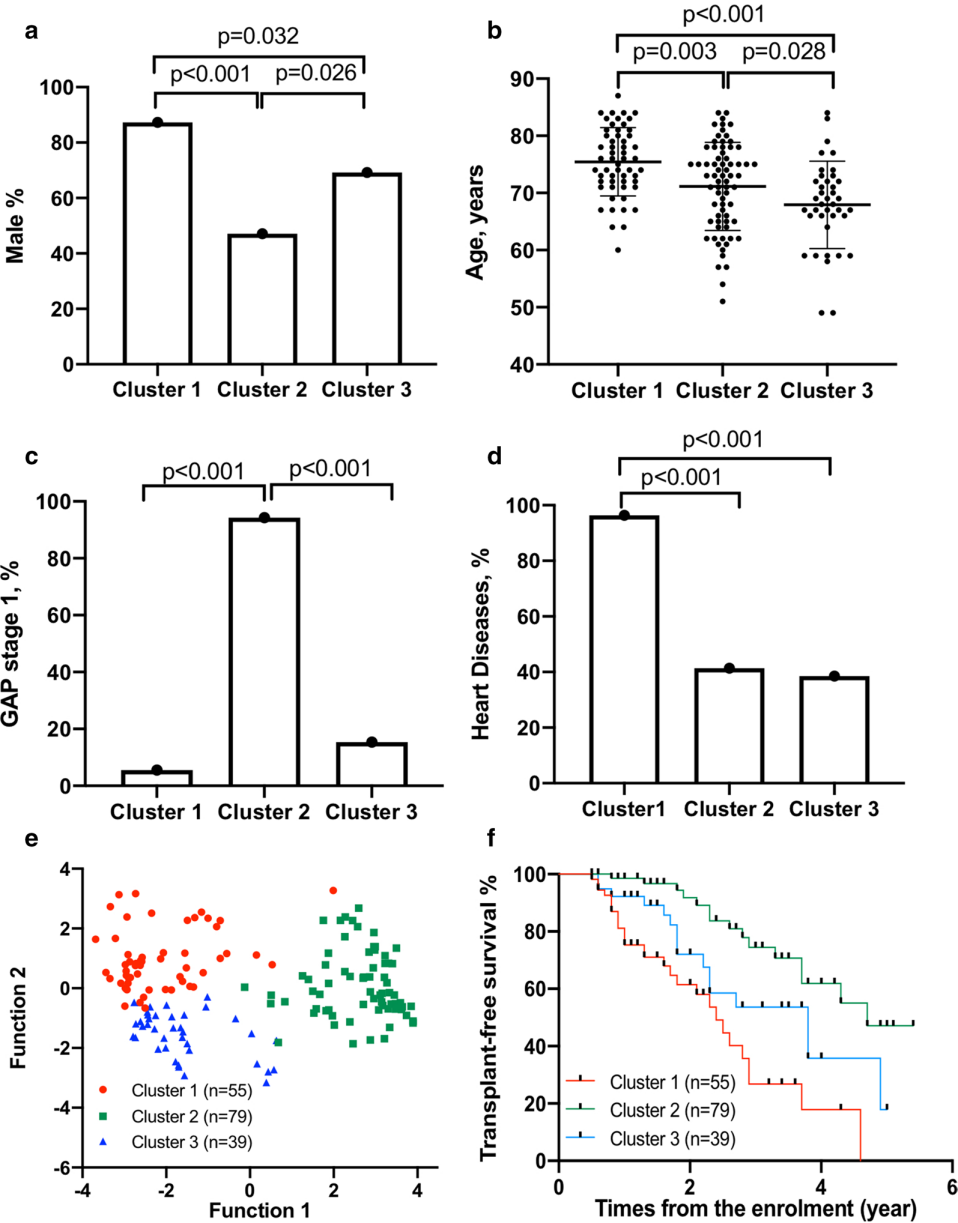
Characteristics of clusters, distribution and survival. In **a**–**d** shown the basic characteristics of clusters; **e** The distribution in clusters, largest absolute correlation between each variable and any discriminant function in Function 1 (GAP stage 1, CPI ≤ 45% TLC ≥ 65%, DLCO ≥ 55%, males, LpSaO2, and 6MWD) and in Function 2 (CCI, the number of comorbidities, heart diseases, FVC ≥ 75%, age, smoking history, acid reflux and BMI); **f** Kaplan–Meier analysis for mortality in clusters

A two-step cluster analysis was performed with 15 variables selected on basis of baseline characteristics and severity (Table 6). Altogether, 164 patients were followed ≥ 6 months after exclusion of patients with missing data. Factor analysis showed the selected variables were suitable for further analysis, since the KMO measure of sampling adequacy was 0.612 and Bartlett's Test of sphericity demonstrated a significant difference (p < 0.001). Three clusters were identified in Fig. 4 A-D; patients in cluster 1 (n = 55) consisted mostly of heart diseases (96.4%), mainly male patients (87.3%) with moderate-severe disease at baseline; Cluster 2 (n = 70) was characterized by mild disease with more than 50% females and few comorbidities; Cluster 3 (n = 39) were younger, moderate-severe patients with few comorbidities. The discriminant analysis showed function 1 to mainly consist of the disease severity variables, while function 2 mainly contained comorbidity variables (Fig. 4e). Kaplan Meier analysis of clusters showed that patients in cluster 1 had a worst survival compared to cluster 2 and 3 (log rank *p* < 0.001 and *p* = 0.036), whereas patients in cluster 2 had the best survival compared to cluster 3 (log rank *p* = 0.017) (Fig. 4f). Multivariable Cox analysis showed that cluster 1 (HR: 3.154, 95%CI (1.855–5.364), *p* < 0.001) and cluster 2 (HR: 0.291, 95%CI (0.160–0.528, *p* < 0.001)) were predictors of survival, after adjustment of anti-fibrotic use.

**Table 6:** The characteristics of clusters in SIPFR

Variables	Cluster			*P*-value
	1 (n:55)	2 (n:79)	3 (n:39)	
A				
Age, years, at enrolment	73 (72–80)	73 (65–77)	68 (66–72)	< 0.001
BMI, kg·m^2^	26.9 (25.0–28.6)	25.8 (23.6–30.8)	27.7 (24.8–30.6)	0.575
Male (yes, %)	48 (87.3)	33 (47.1)	27 (69.2)	< 0.001
Smoking history, (yes, %)	44 (80)	53 (75.7)	26 (66.7)	0.333
6MWD, m	397 (296–464)	354 (398–516)	415 (377–474)	0.002
LpSaO2, %	87 (82–90)	90 (87–93)	84 (81–90)	< 0.001
Comorbidities				
The number of comorbidities	3 (2–3)	1 (0–2)	1 (1–1)	< 0.001
CCI	6 (5–7)	4 (3–5)	4 (3–4)	< 0.001
Acid reflux, (yes, %)	24 (43.6)	21 (30)	13 (33.3)	0.273
Heart diseases, (yes, %)	53 (96.4)	29 (41.4)	15 (38.5)	< 0.001
Severity				
FVC% predicted ≥ 75%, (n, %)	23 (41.8)	56 (80)	2 (5.1)	< 0.001
DLCO predicted ≥ 55%, (n, %)	9 (16.4)	46 (65.7)	6 (15.4)	< 0.001
TLC predicted ≥ 65%, (n, %)	19 (34)	62 (88.6)	12 (30.8)	< 0.001
CPI ≤ 45%, (n, %)	12 (21.8)	62 (88.6)	2 (5.1)	< 0.001
GAP stage 1, (n, %)	3 (5.5)	66 (94.3)	6 (15.4)	< 0.001

Data are presented as median (25th percentile-75th percentile) unless otherwise indicated. *IPF* idiopathic pulmonary fibrosis, *BMI* body mass index, *CCI* Charlson Comorbidity Index, *FVC* forced vital capacity, *DLCO* diffusing capacity of carbon monoxide; *TLC* total lung capacity, *GAP* gender-age-physiology index for IPF, *K-BILD* King's brief interstitial lung disease health status questionnaire, *6MWD* 6 min walking distance during 6MWT, *L-SpO*_*2*_ Lowest oxygen saturation during 6MWT

For validation, we carried out discriminant analysis by the leave-one-out method to ensure stability and repeatability of the model. This method showed that 95.7% of the originally grouped cases were correctly classified, and 90.9% of the cross-validated grouped cases were correctly classified.

## Discussion

Similar to other IPF-registries, we demonstrate a heterogeneous patient cohort with respect to age, disease severity, and co-morbidities. The cumulative 1, 2, 3, 4 and 5 year mortality was 7, 16, 30, 39 and 48%, respectively. We were able to confirm that lung function, 6MWD and BMI are significant predictors of mortality [17, 18, 24, 30]. Patients receiving anti-fibrotic therapy had better survival than untreated patients in all and in GAP stage above 1. We investigated the agreement of the GAP stage with single and composite measures of physiological impairment and found that patients with mild physiological impairment have better survival than patients with moderate-severe disease. Three clusters were identified of which one, consisting of males with heart diseases, multiple comorbidities, and high GAP stage, had the worst survival.

One of the important findings in this study is a stratification for a standardized approach to disease severity. Potential stratifications of disease severity have been a widely discussed topic in the community for a long time. Heterogeneity in IPF is multidimensional. Although it is difficult to define the "best" definition of disease stratification, classification requires consideration of these disparate domains. Some of these characteristics have been incorporated in indexes of different domains such as the GAP-index and the composite physiological index, CPI. Patient registries give us the opportunity to include a heterogeneous group of patients with wide ranges of baseline physiology and disease severity. Our results showed that CPI ≤ 45, DLCO ≥ 55%, FVC ≥ 75%, and TLC ≥ 65%, agreed well with GAP stage 1 for staging of mild physiological impairment. This was a first study to define the mild physiological impairment by TLC% in a large scale of IPF patients. Moreover, we also showed that the presence of mild impairment at baseline was predictive of better survival compared to patients with moderate-severe disease on univariable as well as multivariable Cox analysis adjusting for age, gender, BMI, smoking status, and anti-fibrotic use.

To the best of our knowledge, no cluster analysis has been done on the IPF registry cohorts using longitudinal data so far. We report an explorative analysis of potential phenotypes of IPF patients in SIPFR, including our newly defined "mild" IPF classification, comorbidities, and demographic data. More than 40% of the patients had two or three comorbidities. Although we did not find a significant association between comorbidities and outcome in the univariate analysis, comorbidities showed high predictor importance in the cluster analysis. This may reflect the real-world IPF patient since single or composite variables have some limitations as disease predictors. Three clusters were identified, with GAP, comorbidities, and gender deemed important factors between clusters. Heart diseases and severity factors had high predictor importance value in our cluster analysis. As shown in other studies, IPF and heart disease may share several risk factors, and IPF has been associated with atherosclerosis [31–33]. The cluster comprising moderately to severe diseased males with heart diseases had worst survival, and mild disease cluster with less comorbidities had best survival. Thus, phenotypes may offer a novel multidimensional approach for predicting outcomes of patients with IPF and suggest patients’ need for special management.

Registries provide the opportunity to study disease progression in patients with anti-fibrotic treatment [10, 30]. In Sweden, anti-fibrotic drugs are completely reimbursed, which results in a large number of patients being on treatment [34]. Thus, approximately 65% of the patients received anti-fibrotic treatment in our study, which is considerably more than in Germany (44%) [9], Finland (26%) [10], and Australia (23%) [6]. The present study shows that patients on anti-fibrotic therapy appear to survive longer than untreated patients, a result similar to what other registries have reported [6, 8, 20]. In order to avoid a potential bias in mortality analysis, we showed that there were no significant differences in baseline lung function between anti-fibrotic treated and untreated groups. Furthermore, we adjusted the potential confounders (age, gender, BMI, smoking history, and anti-fibrotic use) at baseline to identify the association between low lung function parameters and mortality. The curves of antifibrotic use and untreated could be clearly distinguished in the Cox model. Although the effect driven by lung function decline was not included in the current baseline project, it is a focus in an upcoming project.

Interestingly, twenty-two patients had been followed ≥ 6 months, who received the switched antifibrotics treatment (Table 4). Reasons for switching antifibrotics is not a dedicated variable in the registry, resulting in a risk of missing disease progression as a cause to the switch. This might be the case for some of these patients in our dataset. In our experience, side effects make up the main reason and are reported for some of the patients in this group. Disease progression is, in our experience, a minor reason for switching treatment, simply because there are no defined definition of stable or progressive disease when it comes to the individual patient. It is important to clarify that our registry, like all other registries, is not designed to compare treatment effects. Differences in characteristics of the compared groups, non-randomization, other undetected confounders, and missing registry data are important factors that require a cautious interpretation of these results. For the purpose of studying treatment effects, well-powered randomized controlled trials are the only gold standard. Thus, lack of improved survival, or survival benefits, does not imply the absence or presence of a true, underlying difference between the groups. The favourable effect on survival of the “switched group” may only be hypothesis-generating and interpreted with caution since pirfenidone and nintedanib have different mechanisms of action. The idea of sequential treatment strategies in IPF has been discussed before, with few retrospective studies on small cohorts, supporting such strategy [35–37].

A number of limitations are worth noting. Firstly, no estimates were made for missing categorical and continuous data and missing data was not involved in further analysis. Secondly, while other studies and registries have highlighted the poorer prognosis in patients with pulmonary hypertension and/or lung cancer, our registry does not collect that type of data, potentially missing other explanatory variables for prognosis. Thirdly, prevalent patients, consisting of 35%, may have a slower disease progression [10, 11], increasing the risk of bias in the survival analysis. Finally, we considered the two timelines from diagnosis and enrolment and adjusted the confounders, but residual confounding might be possible and may have affected the regression analysis [38]. In addition, the effect of smoking on IPF behaviour was not deeply analysed, due to the small numbers of current smokers (n = 24). Only 19 of 24 (4%) patients had been followed ≥ 6 months. Hence, only smoking history (ex-and current smokers) were evaluated in this study. The influence of current smoking on the disease will require a larger cohort. Potential preventive effects of antifibrotics on hospitalizations and exacerbations and thus also on mortality were not analysed in this paper. Currently, data related to exacerbations in the Swedish IPF-registry is limited and needs further distinguishment and collection (e.g. distinguishing hospitalizations related to comorbidities from IPF related exacerbations).

## Conclusion

We conclude that both disease severity and phenotype are closely associated with outcome in IPF which may be important for disease behaviour and follow-up. Survival was significantly higher in IPF patients with anti-fibrotic therapy, especially in patients with moderate-severe disease. Mild physiological impairments could be defined by TLC ≥ 65% in SIPFR. IPF patients with mild physiological impairment have better survival than patients with moderate-severe disease. Phenotypes may contribute to predicting outcomes of patients with IPF and suggest the patients’ need for special management, whereas single or composite variables have some limitations as disease predictors. Our results provide an insight into the characteristics, management, and outcome of IPF-patients in real life.

## Role of the sponsors

The study funders/sponsors had no role in this study, including the design, collection, management, analysis, writing, review, or approval of the manuscript; and decision to submit the manuscript for publication.

## Data Availability

No.

## Abbreviations

AIPFR: The Australian idiopathic pulmonary fibrosis registry
BIC: The Schwarz Bayesian Criterion
BMI: Body mass index
CCI: The Charlson Comorbidity Index
COPD: Chronic obstructive pulmonary disease
CPI: Composite physiological index
DLCO%: Diffusing capacity of carbon monoxide, % of predicted
EurIPFreg: European idiopathic pulmonary fibrosis registry
FEV1: Forced expiratory volume in 1 s
FEV1%: Forced expiratory volume in 1 s, % of predicted
FinnishIPF: The Finnish idiopathic pulmonary fibrosis registry
FVC: Forced vital capacity
FVC%: Forced vital capacity, % of predicted
GAP: Gender-age-physiology index for idiopathic pulmonary fibrosis
IPF: Idiopathic pulmonary fibrosis
IQR: Interquartile ranges (IQR)
INSIGHTS: The German investigating significant health trends in idiopathic pulmonary fibrosis registry
k: Kappa value
K-BILD: King's brief interstitial lung disease health status questionnaire
KMO: The Kaiser–Meyer–Olkin (KMO) value of the scale
LC: The registry coordinator
L-SpO_2_: Lowest oxygen saturation during 6-min walking test
SIPFR: The Swedish idiopathic pulmonary fibrosis registry
TLC %: Total lung capacity, % of predicted
QoL: Quality of life
6MWD: 6-Min walking distance
6MWT: 6-Min walking test
UIP: Usual interstitial pneumonia

## References

[1] Guenther A, Krauss E, Tello S, Wagner J, Paul B, Kuhn S, Maurer O, Heinemann S, Costabel U, Barbero MAN (2018). The European IPF registry (eurIPFreg): baseline characteristics and survival of patients with idiopathic pulmonary fibrosis. Respir Res.

[2] Skold CM, Bendstrup E, Myllarniemi M, Gudmundsson G, Sjaheim T, Hilberg O, Altraja A, Kaarteenaho R, Ferrara G (2017). Treatment of idiopathic pulmonary fibrosis: a position paper from a Nordic expert group. J Intern Med.

[3] Martinez FJ, Chisholm A, Collard HR, Flaherty KR, Myers J, Raghu G, Walsh SL, White ES, Richeldi L (2017). The diagnosis of idiopathic pulmonary fibrosis: current and future approaches. Lancet Respir Med.

[4] Sgalla G, Iovene B, Calvello M, Ori M, Varone F, Richeldi L (2018). Idiopathic pulmonary fibrosis: pathogenesis and management. Respir Res.

[5] Richeldi L, Collard HR, Jones MG (2017). Idiopathic pulmonary fibrosis. Lancet.

[6] Jo HE, Glaspole I, Grainge C, Goh N, Hopkins PM, Moodley Y, Reynolds PN, Chapman S, Walters EH, Zappala C (2017). Baseline characteristics of idiopathic pulmonary fibrosis: analysis from the Australian Idiopathic Pulmonary Fibrosis Registry. Eur Respir J.

[7] Jo HE, Glaspole I, Moodley Y, Chapman S, Ellis S, Goh N, Hopkins P, Keir G, Mahar A, Cooper W (2018). Disease progression in idiopathic pulmonary fibrosis with mild physiological impairment: analysis from the Australian IPF registry. BMC Pulm Med.

[8] Behr J, Prasse A, Wirtz H, Koschel D, Pittrow D, Held M, Klotsche J, Andreas S, Claussen M, Grohe C (2020). Survival and course of lung function in the presence or absence of antifibrotic treatment in patients with idiopathic pulmonary fibrosis: long-term results of the INSIGHTS-IPF registry. Eur Respir J.

[9] Behr J, Kreuter M, Hoeper MM, Wirtz H, Klotsche J, Koschel D, Andreas S, Claussen M, Grohe C, Wilkens H (2015). Management of patients with idiopathic pulmonary fibrosis in clinical practice: the INSIGHTS-IPF registry. Eur Respir J.

[10] Kaunisto J, Salomaa ER, Hodgson U, Kaarteenaho R, Kankaanranta H, Koli K, Vahlberg T, Myllarniemi M (2019). Demographics and survival of patients with idiopathic pulmonary fibrosis in the FinnishIPF registry. ERJ Open Res.

[11] Tran T, Sterclova M, Mogulkoc N, Lewandowska K, Muller V, Hajkova M, Kramer MR, Jovanovic D, Tekavec-Trkanjec J, Studnicka M (2020). The European MultiPartner IPF registry (EMPIRE): validating long-term prognostic factors in idiopathic pulmonary fibrosis. Respir Res.

[12] Zurkova M, Kriegova E, Kolek V, Lostakova V, Sterclova M, Bartos V, Doubkova M, Binkova I, Svoboda M, Strenkova J (2019). Effect of pirfenidone on lung function decline and survival: 5-yr experience from a real-life IPF cohort from the Czech EMPIRE registry. Respir Res.

[13] Ferrara G, Carlson L, Palm A, Einarsson J, Olivesten C, Skold M (2016). R SIPF: Idiopathic pulmonary fibrosis in Sweden: report from the first year of activity of the Swedish IPF-Registry. Eur Clin Res J.

[14] Wuyts WA, Dahlqvist C, Slabbynck H, Schlesser M, Gusbin N, Compere C, Maddens S, Kirchgaessler KU, Bartley K, Bondue B (2018). Baseline clinical characteristics, comorbidities and prescribed medication in a real-world population of patients with idiopathic pulmonary fibrosis: the PROOF registry. BMJ Open Respir Res.

[15] Wuyts WA, Dahlqvist C, Slabbynck H, Schlesser M, Gusbin N, Compere C, Maddens S, Lee YC, Kirchgaessler KU, Bartley K, Bondue B (2019). Longitudinal clinical outcomes in a real-world population of patients with idiopathic pulmonary fibrosis: the PROOF registry. Respir Res.

[16] Latsi PI, du Bois RM, Nicholson AG, Colby TV, Bisirtzoglou D, Nikolakopoulou A, Veeraraghavan S, Hansell DM, Wells AU (2003). Fibrotic idiopathic interstitial pneumonia—the prognostic value of longitudinal functional trends. Am J Respir Crit Care Med.

[17] Mogulkoc N, Brutsche MH, Bishop PW, Greaves SM, Horrocks AW, Egan JJ (2001). Greater Manchester Pulmonary Fibrosis C: Pulmonary function in idiopathic pulmonary fibrosis and referral for lung transplantation. Am J Respir Crit Care Med.

[18] Erbes R, Schaberg T, Loddenkemper R (1997). Lung function tests in patients with idiopathic pulmonary fibrosis. Are they helpful for predicting outcome?. Chest.

[19] Pesonena I, Gao J, Kalafatisb D, Carlsona L, Skold M, Ferrara G (2020). Six-minute walking test outweighs other predictors of mortality in idiopathic pulmonary fibrosis. A real-life study from the Swedish IPF registry. Res Med.

[20] Kalafatis D, Gao J, Pesonen I, Carlson L, Skold CM, Ferrara G (2019). Gender differences at presentation of idiopathic pulmonary fibrosis in Sweden. BMC Pulm Med.

[21] Richeldi L, du Bois RM, Raghu G, Azuma A, Brown KK, Costabel U, Cottin V, Flaherty KR, Hansell DM, Inoue Y (2014). Efficacy and safety of nintedanib in idiopathic pulmonary fibrosis. N Engl J Med.

[22] King TE, Bradford WZ, Castro-Bernardini S, Fagan EA, Glaspole I, Glassberg MK, Gorina E, Hopkins PM, Kardatzke D, Lancaster L (2014). A phase 3 trial of pirfenidone in patients with idiopathic pulmonary fibrosis. N Engl J Med.

[23] Richeldi L (2014). Treatments for idiopathic pulmonary fibrosis. N Engl J Med.

[24] King TE, Tooze JA, Schwarz MI, Brown KR, Cherniack RM (2001). Predicting survival in idiopathic pulmonary fibrosis: scoring system and survival model. Am J Respir Crit Care Med.

[25] Noble PW, Albera C, Bradford WZ, Costabel U, Glassberg MK, Kardatzke D, King TE, Lancaster L, Sahn SA, Szwarcberg J (2011). Pirfenidone in patients with idiopathic pulmonary fibrosis (CAPACITY): two randomised trials. Lancet.

[26] Raghu G, Collard HR, Egan JJ, Martinez FJ, Behr J, Brown KK, Colby TV, Cordier JF, Flaherty KR, Lasky JA (2011). An official ATS/ERS/JRS/ALAT statement: idiopathic pulmonary fibrosis: evidence-based guidelines for diagnosis and management. Am J Respir Crit Care Med.

[27] Skold CM. Idiopatisk lungfibros, vårdprogram, Swedish Respiratory Society [Internet]. 2019. http://slmf.se/wp-content/uploads/2019/03/vp_ipf_19_web.pdf. Accessed 1 Feb 2021.

[28] Racanelli AC, Kikkers SA, Choi AMK, Cloonan SM (2018). Autophagy and inflammation in chronic respiratory disease. Autophagy.

[29] Ilmarinen P, Tuomisto LE, Niemela O, Tommola M, Haanpaa J, Kankaanranta H (2017). Cluster analysis on longitudinal data of patients with adult-onset asthma. J Allergy Clin Immunol Pract.

[30] Ley B, Ryerson CJ, Vittinghoff E, Ryu JH, Tomassetti S, Lee JS, Poletti V, Buccioli M, Elicker BM, Jones KD (2012). A Multidimensional index and staging system for idiopathic pulmonary fibrosis. Ann Intern Med.

[31] Oldham JM, Collard HR (2017). Comorbid conditions in idiopathic pulmonary fibrosis: recognition and management. Front Med (Lausanne).

[32] Nathan SD, Basavaraj A, Reichner C, Shlobin OA, Ahmad S, Kiernan J, Burton N, Barnett SD (2010). Prevalence and impact of coronary artery disease in idiopathic pulmonary fibrosis. Respir Med.

[33] Hubbard RB, Smith C, Le Jeune I, Gribbin J, Fogarty AW (2008). The association between idiopathic pulmonary fibrosis and vascular disease a population-based study. Am J Respir Crit Care Med.

[34] Pesonen I, Carlson L, Murgia N, Kaarteenaho R, Skold CM, Myllarniemi M, Ferrara G (2018). Delay and inequalities in the treatment of idiopathic pulmonary fibrosis: the case of two Nordic countries. Multidiscip Respir Med.

[35] Wuyts WA, Antoniou KM, Borensztajn K, Costabel U, Cottin V, Crestani B, Grutters JC, Maher TM, Poletti V, Richeldi L (2014). Combination therapy: the future of management for idiopathic pulmonary fibrosis?. Lancet Respir Med.

[36] Vianello A, Salton F, Molena B, Turato C, Graziani ML, Braccioni F, Frassani V, Sella D, Pretto P, Paladini L (2020). Nintedanib treatment for idiopathic pulmonary fibrosis patients who have been switched from pirfenidone therapy: a retrospective case series study. J Clin Med.

[37] Milger K, Kneidinger N, Neurohr C, Reichenberger F, Behr J (2015). Switching to nintedanib after discontinuation of pirfenidone due to adverse events in IPF. Eur Respir J.

[38] O'Brien EC, Hellkamp AS, Neely ML, Swaminathan A, Bender S, Snyder LD, Culver DA, Conoscenti CS, Todd JL, Palmer SM (2020). Disease severity and quality of life in patients with idiopathic pulmonary fibrosis: a cross-sectional analysis of the IPF-PRO registry. Chest.

